# Complete genome and methylome analysis of *Neisseria meningitidis* associated with increased serogroup Y disease

**DOI:** 10.1038/s41598-020-59509-y

**Published:** 2020-02-27

**Authors:** Bianca Stenmark, Odile B. Harrison, Lorraine Eriksson, Brian P. Anton, Alexey Fomenkov, Richard J. Roberts, Ave Tooming-Klunderud, Holly B. Bratcher, James E. Bray, Sara Thulin-Hedberg, Martin C. J. Maiden, Paula Mölling

**Affiliations:** 1 Department of Laboratory Medicine, Faculty of Medicine and Health, Örebro University, Örebro, Sweden; 2 Department of Zoology, University of Oxford, Oxford, United Kingdom; 3 Centre for Ecological and Evolutionary Synthesis, Department of Biosciences, University of Oslo, Oslo, Norway; 4 New England Biolabs, Ipswich, Massachusetts USA

**Keywords:** Clinical microbiology, Microbial genetics

## Abstract

Invasive meningococcal disease (IMD) due to serogroup Y *Neisseria meningitidis* emerged in Europe during the 2000s. Draft genomes of serogroup Y isolates in Sweden revealed that although the population structure of these isolates was similar to other serogroup Y isolates internationally, a distinct strain (YI) and more specifically a sublineage (1) of this strain was responsible for the increase of serogroup Y IMD in Sweden. We performed single molecule real-time (SMRT) sequencing on eight serogroup Y isolates from different sublineages to unravel the genetic and epigenetic factors delineating them, in order to understand the serogroup Y emergence. Extensive comparisons between the serogroup Y sublineages of all coding sequences, complex genomic regions, intergenic regions, and methylation motifs revealed small point mutations in genes mainly encoding hypothetical and metabolic proteins, and non-synonymous variants in genes involved in adhesion, iron acquisition, and endotoxin production. The methylation motif C**A**CNNNNN**T**AC was only found in isolates of sublineage 2. Only seven genes were putatively differentially expressed, and another two genes encoding hypothetical proteins were only present in sublineage 2. These data suggest that the serogroup Y IMD increase in Sweden was most probably due to small changes in genes important for colonization and transmission.

## Introduction

The Gram-negative encapsulated bacterium *Neisseria meningitidis* is a common commensal found exclusively in the human nasopharyngeal mucosa. It is the leading cause of epidemic meningitis and sepsis^1^. Invasive meningococcal disease (IMD) is mainly caused by meningococci expressing specific capsular groups (i.e. serogroups) and belonging to particular hyperinvasive lineages^2,3^, which have a changing global distribution over time. An increase in IMD due to serogroup Y occurred in the United States in the 1990s, and from the end of the 2000s this was also the case in Europe^4,5^. This serogroup was the most prevalent cause of IMD in Sweden between 2010 and 2015, representing 53% of all IMD in 2015^6^. Characterization by multilocus sequence typing (MLST) and sequencing of the antigens FetA, FHbp, PenA, PorA, and PorB, revealed that three serogroup Y strain types were responsible for IMD in Sweden, in particular those with the genotype Y: P1.5-2, 10-1: F4-1: ST-23 clonal complex 23 (cc23) along with PorB allele 3–36, FHbp allele 25, and PenA allele 22, referred to as strain YI^7^. Illumina whole genome sequencing (WGS) of 185 serogroup Y genomes from Sweden showed that the majority of those causing IMD clustered with strain YI, belonging to the WGS lineage 23.1^8^. Analysis of genes core to the meningococcus (cgMLST) revealed that this cluster, although antigenically identical, contained an average of 100 core loci with allelic differences, delineating it into sublineages 1 and 2^8^. Analysis on a limited selection of 177 loci hypothesized to play a role in meningococcal virulence showed that 10 of these loci differed between the two sublineages. Because 213 core loci were incompletely assembled in at least one isolate in the draft genomes, the genetic analysis was only based on 1,241 completely assembled genes. However, the study showed that the temporal distribution of the two sublineages in Sweden coincided with an increase in serogroup Y IMD due to one of the sublineages, appearing in Sweden after 2006.

Single molecule real-time (SMRT) PacBio sequencing also includes data on DNA methylation in the form of N6-methyladenine (m6A), N4-methylcytosine (m4C) and C5-methylcytosine (m5C), only poorly at best. Enzymes that methylate (MTases) are part of the restriction-modification (RM) system, which acts as a defence mechanism against the invasion of foreign DNA in prokaryotes^9^. The restriction endonucleases (REases) cleave double stranded DNA with specific patterns, and methyl groups are added by MTases to specific motifs in order to prevent degradation by the REases. There are four types of RM systems, three of which have been found in *Neisseria*^10,11^. Type I systems consist of three subunit proteins: R (restriction), M (modification), and S (specificity). Type II systems consist of individual RM enzymes that bind to and cleave at the same position, or close to that sequence. Type III systems are composed of two protein subunits, Mod and Res that recognize non-palindromic motifs^12^. Although DNA methylation is best known for its role in prokaryotic defence, and is important for genetic flux, it has also been shown to have roles in gene expression^13,14^, DNA replication initiation^15,16^, virulence^17^ and are often phase variable^11^.

The aim of this study was to robustly identify all genetic alterations and differences in methylation between sublineage 1 (responsible for the serogroup Y increase in Sweden) and sublineage 2 using high-quality PacBio-derived finished genomes, thereby enhancing our understanding of how invasive meningococci may emerge over time.

## Materials and Methods

### Isolate collection and genome sequencing

**Figure 1:**
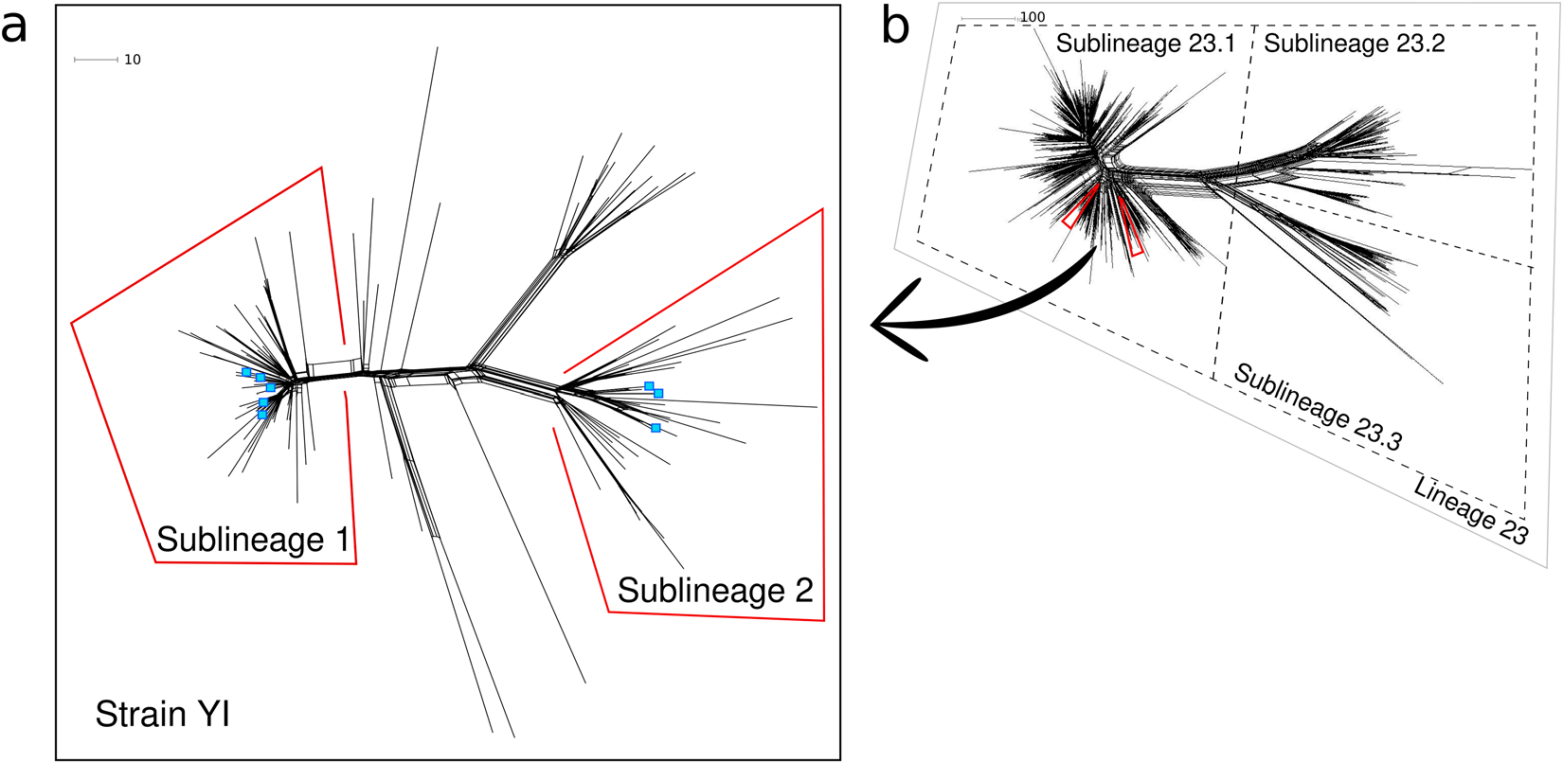
Neighbour-net network based on the comparison of 1,605 *Neisseria meningitidis* core genome loci. Panel a shows strain YI from Sweden subclustered into sublineage 1 and sublineage 2^8^. These two sublineages belong to sublineage 23.1^8^, as shown in panel b of all clonal complex 23 genomes in the pubMLST *Neisseria* database (n = 1,576; accessed 20/01/10). Isolates from the present study are marked in blue. Isolate IDs for isolates belonging to sublineages 1 (n = 59) and 2 (n = 32) are provided in Supplementary Table 1. The scale bars denote the number of loci with allelic differences. This figure was generated using SplitsTree v 4.15.1 (http://www.splitstree.org/) and subsequently exported to Inkscape v0.92 (https://inkscape.org/) for additional edits.

Eight isolates belonging to YI sublineages 1 and 2 were chosen based on the phylogenetic clustering generated using Illumina-derived WGS of serogroup Y isolates from Sweden between 1995 and 2012^8^ (Fig. 1). All isolates had the following designation: Y: P1.5-2,10-1,36-2: F4-1: ST-23 (cc23), and had PorB allele 3–36, FHbp allele 25, and PenA allele 22, except for isolate 98–182: FHbp allele 276, isolate 11–14: PorB allele 3–117, and isolate 12–176: PorA VR2 10–85. Sublineage 1 (n = 59) and sublineage 2 (n = 32) HiSeq genomes^8^ were used to confirm differences in presence/absence and premature stop codons of genes delineating the two sublineages. PubMLST *Neisseria* database identification numbers are shown in Supplementary Table 1.

Isolates were cultured on chocolate agar at 37 °C in a 5% CO_2_ enriched atmosphere overnight and archived at −70 °C. Genomic DNA was extracted using the Wizard Genomic DNA purification kit (Promega) according to the manufacturer’s instructions. Libraries were prepared using the Pacific Biosciences 10 kb library preparation protocol. Size selection of the final libraries was performed using BluePippin (Sage Science) or AMPure PB beads (PacBio). The libraries were sequenced on a Pacific Biosciences RS II sequencer using P4-C2 or P6-C4 chemistry with 1–3 SMRT cells each. One of the genomes (12–221) was sequenced with >1000x coverage in order to detect m5C motifs.

### Genome assembly and annotation

Reads were assembled *de novo* using HGAP v3 (Pacific Biosciences, SMRT Analysis Software v2.3.0, smrtanalysis_2.3.0.140936.p5.167094). Sequencing and assembly metrics are shown in Supplementary Table 2. Quiver^18^ was used to correct sequencing errors in the assemblies by mapping the raw reads back to the PacBio assembly. Mimimus2 software from the Amos package^19^ was used to circularize the genomes. Illumina HiSeq 2000 100 bp reads from a previous study^8^ (ERR405856, ERR405873, ERR405911, ERR405968, ERR405969, ERR406012, ERR406017, and ERR406024) were filtered at Q30 and mapped onto the genomes obtained from the Pacific Biosciences RS II sequencer using CLC Genomics Workbench v8.0.1 (Qiagen) to detect any assembly and sequencing errors. To strengthen any corrections made, PacBio subreads were mapped onto the PacBio assemblies. All errors with >50% mapping frequency of the HiSeq reads and 9–28% of the mapped PacBio reads in agreement with the HiSeq reads were manually corrected. Thirty errors were found using these criteria; twenty-nine of the 30 corrections were single nucleotide insertions in homopolymeric tracts (5–9 nucleotides long) and one was in a repetitive region (Supplementary Table 3). The chromosome was adjusted so that the first base was upstream of the *dnaA* gene.

PacBio assemblies were annotated in two ways: (i) using the rapid bacterial annotation software Prokka^20^ and (ii) using the PubMLST *Neisseria* database (http://pubMLST.org/neisseria/) where genome data have been deposited. Genes were labelled using the locus tag prefix “NEIS”. NEIS identifiers were linked to an alias table, enabling cross-linking with Prokka annotations. Alleles were automatically assigned to and annotated with the appropriate allele number within the PubMLST *Neisseria* database when these were a ≥98% match to previously defined loci in the database. New alleles ≤98% identical were manually curated and assigned. Allele frequency was determined using the allele designations field filter when browsing all genomes deposited in the PubMLST *Neisseria* database. All eight genomes have been deposited in NCBI under BioProject number PRJNA386981; the PubMLST *Neisseria* database identification numbers are shown in Table 1.

**Table 1:** Genome metrics of the single molecule real-time sequenced *Neisseria meningitidis* serogroup Y isolates (n = 8).

Isolate	PubMLST ID	Collection year	Sub-lineage	Chromosome size (nt^a^)	CDS^b,c^	tRNAs	rRNAs	GC content (%)
95–134	41337	1995	2	2,165,984	2144	59	12	51.71
98–182	41338	1998	2	2,167,995	2159	59	12	51.70
06–178	41339	2006	1	2,167,920	2150	58	12	51.70
11–7	41340	2011	2	2,157,431	2148	59	12	51.77
11–14	41341	2011	1	2,156,539	2143	58	12	51.75
12–176	41342	2012	1	2,168,615	2150	58	12	51.67
12–221	89521	2012	1	2,167,947	2082	58	12	51.69
12–330	41344	2012	1	2,167,944	2151	58	12	51.69

^a^nt = nucleotide.

^b^CDS **=** coding sequence.

^c^CDS features without a/pseudo or/pseudogene qualifier.

### Genome analyses

Annotated genomes were compared using Artemis^21^, ACT^22^, Mauve^23^, and BRIG^24^. To identify variations in coding sequences (CDS) among sublineages, the Genome Comparator Tool, available in the PubMLST *Neisseria* database, was employed using a sublineage as a reference as described previously^25^. Briefly, the Genome Comparator Tool compares genomes using any number of predefined loci in the database or a reference genome. For each locus, the allele sequences, designated by allele numbers, are compared and used to generate a distance matrix based on the number of variable loci across a genome. Distance matrices can subsequently be visualized using the neighbour-net algorithm^26^. The Genome Comparator Tool output includes a list of loci that are: (i) identical; (ii) variable; (iii) missing/absent; and (iv) incomplete (partially present in the genome due to incomplete assembly).

### Core and pan genome

A total of 1,605 loci have been identified as core to meningococci, as they are present in ≥95% *N. meningitidis* isolates (cgMLST, v1.0)^27^. Neighbour-net diagrams were constructed using distance matrices generated by the PubMLST Genome Comparator Tool^27^ and visualized using SplitsTree4^28^. Loci core to the whole genome based lineage 23^27^ were identified through the combined use of Prokka and Genome Comparator. Initially, all loci defined using Prokka were BLAST searched against all of the loci and associated alleles found in the PubMLST *Neisseria* database. This allowed identification of novel loci not yet defined in the PubMLST *Neisseria* database. All novel loci were subsequently verified using Artemis to ensure the correct start and stop codons had been annotated. These were then compared using Genome Comparator in all MLST cc23 isolates deposited in the PubMLST *Neisseria* database (978 cc23 isolates at the time of the study). A whole genome based lineage 23 core and pan genome scheme was then generated.

### Methylation motifs

The RS_Modification_and_Motif_Analysis pipeline analysis platform SMRT Portal (Pacific Biosciences, SMRT Analysis Software v2.3.0, smrtanalysis_2.3.0.140936.p5.167094) was used for genome-wide analysis of modified motifs with quality value (QV) limit > 60. DNA methyltransferase genes associated with the different methyltransferase recognition motifs identified were searched using SEQWARE routines as described previously^29^, and deposited in the Restriction Enzyme Database REBASE^30^. Motif summary files have been deposited in the NCBI submission PRJNA386981 (see Supplementary Table 4 for a summary).

### Enzymatic cleavage with MspJI and FspEI

MspJI and its homologue FspEI (New England Biolabs) was used to enzymatically verify the activity of predicted m5C methylations. These enzymes cleave at a fixed distance from the top-strand m5C (12 or 16 bases), leaving a 4-base 5′ overhang, and the resulting fragments were sequenced to determine the recognition site^31^. MspJI and FspEI cleavage was performed as previously described^31^. In short, 0.5 µg genomic DNA was digested with MspJI and FspEI (New England Biolabs) according to the manufacturer's instructions, and then separated on a 20% polyacrylamide gel electrophoresis (PAGE) in 0.5x TBE buffer and stained with SYBR GOLD. The 30–35 bp gel fragments were excised and purified using the NEB Monarch Nucleic Acid Purification Kit (New England Biolabs). Libraries were prepared for sequencing using the NEBNext Fast DNA Library Prep Set (New England Biolabs) according to the manufacturer’s instructions, except excluding the size selection and running only 12 cycles of PCR. The bioinformatics analysis was performed as previously described^31^.

## Results

### General genome features and comparative genomics

**Figure 2:**
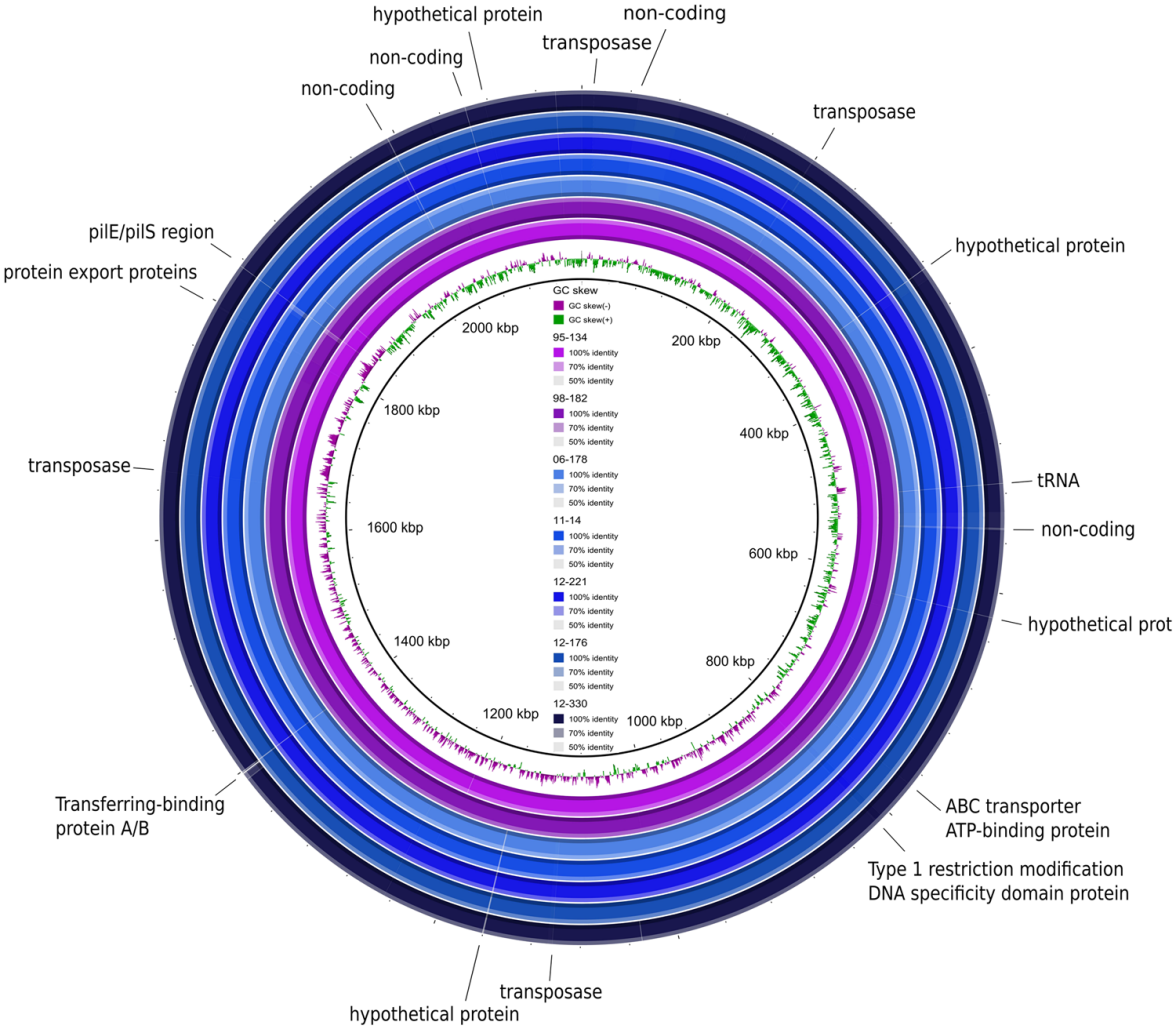
BLAST ring comparing *N. meningitidis* sublineages 1 (in blue; n = 5) and 2 (in purple; n = 2), with isolate 11–7 (sublineage 2) used as reference. The innermost circle shows the GC skew. The protein function of coding regions with low identity between the sublineages are indicated on the outermost circle. This figure was generated using BRIG v 0.95 (http://brig.sourceforge.net/) and subsequently exported to Inkscape v0.92 (https://inkscape.org/) for additional edits.

Eight *N. meningitidis* serogroup Y genomes were sequenced using PacBio RS II and each assembled into a single contiguous sequence. The median genome length was 2,167,932 nucleotides and the median number of predicted CDS was 2,149 (Table 1). The genomes had similar structure and gene synteny (Supplementary Figure 1), with dissimilarities between sublineages found mainly in transposases, hypothetical proteins and non-coding intergenic regions (IGRs) located between genes involved in metabolism, methyltransferases, and prophages (Fig. 2).

**Figure 3:**
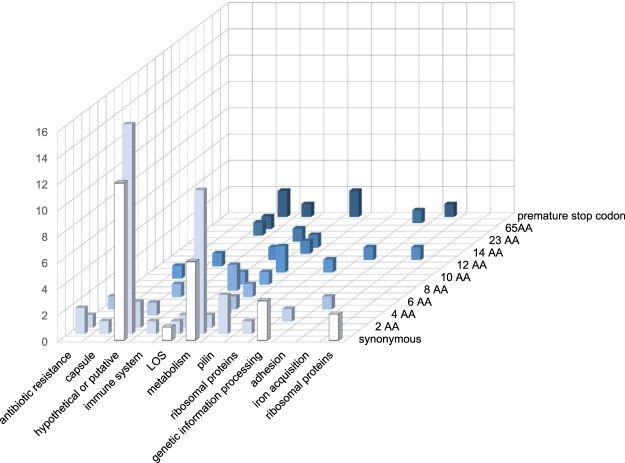
Functions of loci that contained allelic differences resulting in synonymous or non-synonymous changes distinguishing the two sublineages. AA = amino acid change. Classification is according to the scheme in the PubMLST *Neisseria* database^27^. This figure was generated using Microsoft Excel 2016.

Gene-by-gene comparisons of all CDS identified allelic differences in 97 loci between isolates in the two sublineages (Fig. 3 and Supplementary Table 5). Of these loci, 73 had non-synonymous differences, including genes implicated in: adhesion (*opcA*); lipooligosaccharide (LOS) production (*galE*, *galE2*, *lgtA*, *lgtB*, *lot*, and *yhbG*); type IV pili production (*pilI*, *pilQ*, and *pilX*); and iron acquisition (NEIS0669). CRISPR-associated endonuclease Cas1 also differed in one amino acid residue (188 V→A) between sublineages 1 and 2 in all but two isolates among the collection of 91 draft genomes^8^.

Among all CDS in isolates belonging to sublineages 1 and 2, only seven CDS with premature stop codons distinguished the sublineages (Table 2), encoding a hypothetical protein, DNA repair protein, metabolic protein, a RM protein, and inner and outer membrane proteins. Two of these were due to phase variability: NEIS2198 (*opcA*) and NEIS2535. OpcA, which is involved in adhesion to epithelial and endothelial cells important in the infection process^32,33^, has been shown to be phase variable and regulated by homopolymeric cytidine residues in the promoter sequence; ≤ 10 or ≥ 15 cytidine residues suppress expression, 12–13 cytidine residues lead to efficient expression, and 11 or 14 lead to intermediate expression^34^. Sublineage 2 isolates had ≤ 8 contiguous C residues and sublineage 1 isolates had ≥ 14, putatively leading to *opcA* being phase variable off in to both sublineages. Moreover, sublineage 1 had an insertion transposase inserted in NEIS2535 encoding the Type I restriction system specificity protein determining the specificity of the restriction and the modification reactions.

**Table 2:** Presence/absence of genes or genes with premature stop codons delineating sublineage 1 and 2.

Locus tag	Product/function	Size (bp^a^)	Presence in^b^	
			sublineage 1 (frequency)	sublineage 2 (frequency)
NEIS0627	Hypothetical protein	117	Absent (59/59)	Present (32/32)
NEIS1059	Hypothetical protein	606	Stop codon (58/59)	Present (32/32)
NEIS1174	DNA repair protein RadC	714	Stop codon (59/59)	Present (32/32)
NEIS1965	Putative inner membrane transport protein	825–846	Stop codon (59/59)	Present (32/32)
NEIS2535^e^	Type I restriction-modification system S protein	1158–1203	Stop codon^c^ (59/59)	Present (29/32)
NEIS2479	Putative membrane protein	906–1017	Stop codon^c^ (59/59)	Present (32/32)
NEIS2931	Hypothetical protein	699	Absent (59/59)	Present (31/32)
NEIS1126	ABC transporter ATP-binding protein	1893–1932	Present (57/59)^d^	Stop codon (28/32)
NEIS2198 (*opcA*)^e^	Outer membrane protein	786–824	Present (59/59)	Stop codon (32/32)

^a^bp = base pairs.

^b^Among the sublineage 1 (n = 59) and sublineage 2 (n = 32) HiSeq genomes.

^c^Stop due to insertion of a transposase.

^d^Two isolates had incompletely assembled loci.

^e^Phase variable.

Only two genes were absent when comparing both sublineages. Genes NEIS0627 and NEIS2931 (both encoding hypothetical proteins) were absent in all sublineage 1 isolates but present in all sublineage 2 isolates (Table 2). The frequency of these genes in the larger collection of HiSeq genomes (Supplementary Table 1) is shown in Table 2. NEIS2931 was found to share sequence identity with a cornifin small proline rich (SPR) family protein that is strongly induced during differentiation of human epidermal keratinocytes^35^. NEIS0627 was highly prevalent among the 20,357 genomes deposited in the PubMLST *Neisseria* database (accessed 20/09/2019): only 985 of the 20,357 genomes lacked this locus. These 985 isolates were primarily cc23 isolates (167/985) and non-meningococcal isolates *N. lactamica* and *N. gonorrhoeae* (484/985). NEIS2931 was prevalent in cc23 isolates. Although a difference was observed in the number of genes absent or probably not expressed due to stop codons between sublineages 1 and 2 (7/1988 and 2/1975 respectively), this difference was not statistically significant (p=0.01).

A lineage 23^27^ pan genome was defined containing 1,757 core loci and 24 accessory loci. Accessory loci included genes encoding seven hypothetical proteins, a MafB toxin, and a Type I RM system protein (Supplementary Table 6). Of the 2,452 loci defined in the PubMLST *Neisseria* database, 466 were absent in all eight genomes in the present study, some of which putatively associated with pathogenic interactions (Supplementary Table 7).

### Horizontal gene transfer

There were nine regions in which allelic differences were adjacent (2–5 genes in succession) and therefore putatively a consequence of horizontal gene transfer (HGT). These included genes implicated in capsule synthesis, metabolism, iron acquisition, antibiotic resistance, and LOS (Supplementary Table 5). Alleles from sublineage 1 in the putative HGT region including NEIS0625, NEIS0626, and NEIS0628 were unique to cc23 genomes, the majority of which were from Sweden (> 89%) (accessed 14/12/2017). Another putative HGT event included loci NEIS0667, NEIS0668, NEIS0669, NEIS0671, and NEIS0672, resulting in sublineage 2 isolates containing alleles more commonly found in *Neisseria gonorrhoeae* (> 96% for NEIS0667, NEIS0669, and NEIS0671). Alleles from sublineage 2 in the putative HGT event involving NEIS1901 (*lgtB*), NEIS1902 (*lgtA*), NEIS1903, and NEIS1904 were found in 80, 84, 70, and 80 isolates respectively; 55–64% of these were from Swedish cc23 genomes, and the remainder belonged to cc41/44. Isolate 12–330 (sublineage 1) had the shorter *tbpB* isotype characteristic of ST-11 meningococci^36^. Another three isolates belonging to sublineage 1 from the larger 185-isolate collection from our previous study^8^ had the same shorter *tbpB* isotype. The other sublineage 1 and 2 isolates had isotype II found among meningococci belonging to the hyper-invasive clonal complexes including ST-8, ST-18, ST-32, and ST-41/44 as well as *N. gonorrhoeae* isolates.

### Methylome analysis

**Figure 4:**
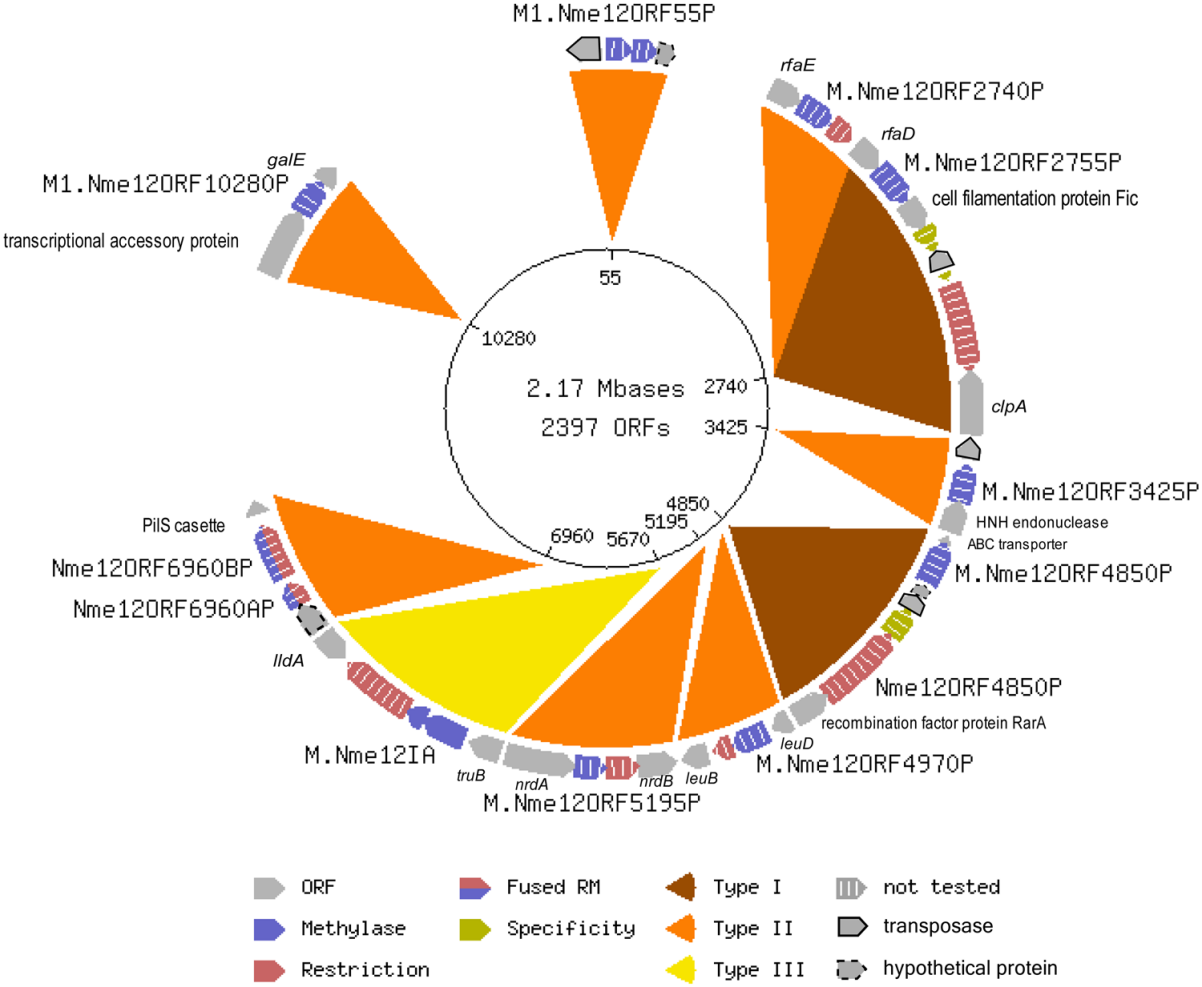
A circular view of the predicted restriction modification (RM) systems in the genome of *N. meningitidis* sublineage 1. ORF = open reading frame. This figure was generated in REBASE (http://rebase.neb.com/rebase/rebase.html) and subsequently exported to Inkscape v0.92 (https://inkscape.org/) for additional edits.

Eleven putative RM systems were identified (putative RM systems for isolate 12–221 are shown in Fig. 4). Initially, the genome-wide analysis of the methylomes identified two m6A and one m5C modified motif: G**A**TC, C**A**CNNNNN**T**AC, and G**G**NN**C**C (Table 3). C**A**CNNNNN**T**AC was found exclusively in isolates belonging to sublineage 2; in sublineage 1, the specificity subunit of the candidate Type I RM system (NEIS2535) was disrupted by a transposase insertion. The motif C**A**CNNNNN**T**AC was only found in one other meningococcal isolate in REBASE, belonging to cc23, suggesting that this may be a cc23 specific motif. Isolate 12–221 was sequenced with higher coverage (1266x) in order to detect m5C motifs that were potentially missed with lower sequencing depth due to the lower effect on delaying the polymerase during PacBio sequencing. Three additional motifs were discovered using higher coverage (Supplementary Table 4). Two of them (CGGCACGCNNNA and CGNGGTAACV) had low signal but were probable m5C motifs; however, no known enzymes could be assigned because the correct motifs could not be determined. The third motif found, a m6A: AC**A**CC, has previously been described as methylated by the MTase encoded by the phase variable gene *modA12*^37^. *modA12* was only putatively expressed by isolates 11–7, the remaining isolates with exception for 12–221 were switched off through translational phase variation by slipped strand mispairing due to a variable number of 5′-AGCC-3′ in the coding region of the gene^38^. Isolate 12–221, belonging to sublineage 1, had one less adenine residue at 1,505 bp resulting in a premature stop codon. This *modA* allele could not be found in any other isolates in the pubMLST *Neisseria* database indicating that this could be the result of a sequencing error. Furthermore, although *modA12* in isolate 11–7 had ON status, the AC**A**CC motif could not be detected in this isolate.

**Table 3:** Putative restriction modification (RM) systems and target motifs found in *N. meningitidis* sublineage 1 (n = 5) and sublineage 2 (n = 3) isolates.

Motif	Detected by	Fraction (%)	RMS Type	Meth type	REBASE entry	Corresponds to NEIS locus	Product	ORF status (frequency)	
								Sublineage 1	Sublineage 2
C**A**CNNNNN**T**AC	PacBio	93–99	I gamma	m6A	M.Nme12ORF4850P	NEIS2535	Type I restriction enzyme system specificity protein	OFF (5/5)^d^	ON (3/3)
–	REBASE		I gamma	m6A	M.Nme12ORF2755P	NEIS2361	NgoAV Type I RM system, DNA methyltransferase subunit M	ON (5/5)	ON (3/3)
G**AT**C	PacBio	94–100	II alpha	m6A	M1.Nme12ORF55P	NEIS0327 (*dam*)	DNA adenine methylase	ON (5/5)	ON (3/3)
GATC	REBASE		II beta	m6A	M2.Nme12ORF55P	NEIS0328 (*dpnIIB*)	modification methylase	ON (4/5)	ON (3/3)
–	REBASE		II gamma	m6A	Nme12ORF6960AP	NEIS2524	hypothetical protein	ON (5/5)	ON (3/3)
–	REBASE		II gamma	m6A	Nme12ORF6960BP	NEIS2523	putative methyltransferase	ON (5/5)	ON (3/3)
CCWGG^a^	REBASE		II	m5C	M.Nme12ORF5195P	NEIS2442	DNA cytosine methylase	ON^e^ (5/5)	ON^e^ (3/3)
G**G**NN**C**C^a,b^	PacBio	75–100	II	m5C	M.Nme12ORF4970P	NEIS1180 (*nlaIV*)	DNA modification methylase	ON (5/5)	ON (3/3)
–	REBASE		II	m5C	M.Nme12ORF3425P	NEIS2555	DNA cytosine methylase	ON (5/5)	ON (3/3)
CCAGA^a^	REBASE		II	m5C	M.Nme12ORF2740P	NEIS0771	DNA cytosine methylase	ON (5/5)	ON (3/3)
GGTGA^c^	REBASE		II	m6A	M1.Nme12ORF10280P	NEIS2910	modification methylase	OFF^f^ (5/5)	OFF^f^ (3/3)
–	REBASE		II	m5C	M2.Nme12ORF10280P	NEIS2854	D12 class adenine-specific DNA methyltransferase	ON (5/5)	ON (3/3)
AC**A**CC^b^	PacBio	70	III beta	m6A	M.Nme12IA	NEIS1310 (modA)	Type III RM system methyltransferase (ModA12)	OFF (5/5)	OFF (2/3)

^a^Enzymatically verified as active.

^b^Poorly detected by PacBio, only found in some isolates.

^c^Enzymatically verified as non-active.

^d^Fragmented due to transposase.

^e^Shorter version: 1,011 nt instead of 1,014 nt.

^f^Frameshifted.

Five m5C genes were bioinformatically predicted from the sequences, but only three motifs (G**C**RY**G**C, G**G**NN**C**C, and C**C**A**G**R) were confirmed as methylated using MspJI and FspEI cleavage (Table 3). The apparent C**C**A**G**R motif may be the result of two MTases, one recognizing C**C**W**G**G and the other C**C**A**G**A.

## Discussion

The increase in incidence of IMD caused by serogroup Y meningococci began in the 1990s and late 2000s in North America and Europe, respectively. In the United States, the increase in cc23 serogroup Y IMD was accompanied by an antigenic shift of the three outer membrane proteins: PorA, FetA, and PorB. The most prevalent serogroup Y strain in Sweden possessed the same antigenic profile; however, Illumina WGS analysis resolved this strain further into two distinct sublineages^8^. One of these sublineages was associated with patients with IMD after 2006, resulting in a marked increase in IMD in Sweden. Variations among the Illumina WGS could not be ruled out as being the result of incomplete genomes, and it was therefore not possible to distinguish robustly differences between these two sublineages, particularly in more complex regions such as IGRs, which are abundant in meningococci, or in the presence or absence of genes. In the present study, PacBio sequencing provides a single contiguous sequence for each genome, which were comprehensively annotated and enabled lineage 23 core and pan genomes to be determined.

Comparison of complete PacBio-derived genome sequences from sublineages 1 and 2 identified sequence differences, mostly limited to IGRs, transposases, and genes encoding hypothetical proteins (Fig. 2). Non-synonymous allelic differences were more abundant among genes encoding hypothetical and metabolic proteins but were also found among genes potentially associated with pathogenicity, such as those implicated in adhesion, LOS production, type IV pili production, and iron acquisition^39^. As with genes involved in pathogenicity, metabolic genes undergo high rates of HGT in meningococci^40,41^ and this is the most likely reason that such were dominant among the genes distinguishing the two sublineages. Nevertheless, functional genomic studies on meningococci during colonization and invasion have shown the importance of metabolic adaptation in the interaction with host cells^42^, which suggests that the differences in metabolic genes identified here may also have contributed to the difference in the emergence of sublineage 1. In contrast to the draft genomes generated by Illumina sequencing previously^8^, the complete PacBio genomes enabled comparison of presence and absence of genes. Only two CDS were absent in sublineage 1 but present in sublineage 2, and only seven were putatively differentially expressed due to premature stop codons, two of them through phase variation. Sequences obtained following WGS will be consensus sequences resulting from a population of colonies rather than a single colony. As a result, it is not possible to reliably infer expression and phase on or off status. Nevertheless, a pattern of phase variation was observed between sublineages 1 and 2. The absence of the hypothetical protein NEIS0627 in sublineage 1 was likely the result of HGT, as this locus was situated with other loci associated with HGT. Finally, the allelic similarities in putative HGT regions with other cc23 isolates and in some cases *N. lactamica* and *N. gonorrhoeae* suggests recombination mainly within cc23 but also potentially with other *Neisseria* species.

The present study revealed that sublineage 1 could not express the Type I restriction system specificity protein due to a transposon, which led to a difference in methylation between the two sublineages. RM systems are known to be located adjacently to mobility-related genes such as transposons in order to promote their own survival^43–45^ and truncation of Type I specificity proteins has been previously described^46^. No other motifs or predicted RM systems were associated with a particular sublineage. Four more m5C MTases were predicted than actually detected. The detection of m5C methylation is difficult using PacBio sequencing; however, deeper sequencing coverage did indicate that more m5C motifs were probably present. It is therefore possible that there are additional m5C motifs unique to a specific sublineage, although enzymatic digestions and ORF status of the predicted m5C MTases did not indicate any such association.

Genes specific to the Type I RM system as well as *modB* and *modD*, which encode phase variable DNA methyltransferases involved in the Type III RM system mediating epigenetic regulation^11,38,47,48^ were absent (Supplementary Table 7). *modB* and *modD* have been described to regulate biofilm formation, adherence and invasion of human epithelial cells as well as increased oxidative stress resistance^14^. These results are consistent with previous results showing that the compositions of different RM systems are clade-specific, suggesting that the population structure is dependent on the restriction of gene flow between clades caused by distinct RM systems^49,50^. This in turn suggests that the unique RM system of cc23 isolates consists of a different set of Type I and III RM systems, which will most likely result in a specific DNA methylation pattern unique to this particular cc, as has been shown in previous studies^50^.

In line with a study by Krauland *et al*.^4^, where pyrosequencing was used to complete the whole genomes of two cc23 strains responsible for the serogroup Y increase in the United States, the genomes in the present study lacked genes encoding the TspB, HmbR, NadA proteins, and the meningococcal disease associated (MDA) islands. These gene products are involved in adhesion, iron acquisition, bacterial immune system, endotoxin production, and mobile elements, and have been associated with meningococcal hyperinvasive lineages^38,51–56^. Their absence could therefore explain the particular clinical outcomes such as pneumonia commonly associated with serogroup Y disease^57–60^. Furthermore, CRISPR-associated genes *cas1* and *cas2*, which were found among all cc23 genomes, have previously been shown to be associated with carriage isolates^53^. Notably, other genes were absent in all the serogroup Y genomes investigated in this study (Supplementary Table 7) and in Krauland *et al*.^4^; these have not been directly linked to virulence of the meningococcus, but are involved in RM, iron acquisition, and mobile elements that potentially play a role in pathogenicity. Shared differences were found between the early and late strain type in the Krauland *et al*. study and the sublineages in the present study, namely in the gene *pilI* involved in the type IV pili structure. None of the other differences in antigen profile or iron acquisition/uptake genes separating the early and late strain types in Krauland's study could be found among the two sublineages in this study; this suggests that the non-synonymous changes in type IV pili encoding genes may play an important role in the emergence of these serogroup Y strains.

Because many of the genes previously regarded as virulence genes have been found in commensal *Neisseria* species^38,61^, and the genomes of carrier and invasive strains are very similar^62^, differences in the presence or absence of genes cannot be the sole reason for the emergence of invasive disease. Instead, our results suggest that point mutations in genes involved in host cell interactions have led to a change in adhesion to epithelial cells, which may have improved colonization, in turn increasing transmission and resulting in expansion of this sublineage. This is also supported by a study showing that no significant differences were found in mortality or clinical outcome between the Swedish strain YI sublineages^60^. It is therefore probable that sublineage 1 has lower virulence but higher transmissibility; this may be due to either the genetic differences found in the present study or an immunologically-naïve host population. Increased transmission or carriage in connection with increased incidence of serogroup Y has been shown in the UK^63,64^ and the United States^65^, and preliminary data from an ongoing carriage study in Sweden indicate similar results but remain to be confirmed.

## Conclusions

PacBio sequencing enabled a full comparison of all CDS, complex regions, IGRs, and methylation motifs among isolates belonging to two serogroup Y sublineages. The YI sublineages were distinguished by non-synonymous mutations in genes involved in metabolism, adhesion, iron acquisition, and endotoxin production, as well as differences observed in methylation motifs, which may have played a role in the emergence of sublineage 1. Additional omics approaches including transcriptomics will be needed to study the effects on gene expression.

## Supplementary information

Supplementary Information.
