# Supplementary material for: Complete genome and methylome analysis of Neisseria meningitidis associated with increased serogroup Y disease
Source: Sci Rep. 2020 Feb 27;10:3644. doi: 10.1038/s41598-020-59509-y (PMC7046676; doi:10.1038/s41598-020-59509-y)
Supplement: Supplementary file 1 — Supplementary Information. [file 41598_2020_59509_MOESM1_ESM.pdf]

SUPPLEMENTARY MATERIAL

Complete genome and methylome analysis of *Neisseria meningitidis* associated with increased serogroup Y disease

Bianca Stenmark*[1], Odile B. Harrison[2], Lorraine Eriksson[1], Brian P. Anton[4], Alexey Fomenkov[4], Richard J Roberts[4], Ave Tooming-Klunderud[3], Holly B. Bratcher[2], James E. Bray[2], Sara Thulin-Hedberg[1], Martin C. J. Maiden[2], Paula Mölling[1]

1 Department of Laboratory Medicine, Faculty of Medicine and Health, Örebro University, Örebro, Sweden.

2 Department of Zoology, University of Oxford, Oxford, United Kingdom.

3 Centre for Ecological and Evolutionary Synthesis, Department of Biosciences, University of Oslo, Oslo, Norway.

4 New England Biolabs, Ipswich, Massachusetts, USA.

*Correspondence:

Bianca Stenmark

bianca.stenmark@regionorebrolan.se

Supplementary Table 1. PubMLST isolate IDs of all sublineage 1 (n=59) and sublineage 2 (n=32) HiSeq genomes [8] used to confirm the results from the complete PacBio genomes in the present study.

**Isolate ID**	
**Sublineage 1**	**Sublineage 2**
26123	26065
26124	26087
26126	26117
26141	26106
26143	26204
26147	26081
26151	26076
26153	26074
26155	26131
26156	26146
26158	26130
26160	26069
26161	26171
26163	26247
26165	26104
26166	26154
26167	26068
26168	26071
26173	26085
26174	26152
26177	26132
26178	26077

26179	26172
26180	26112
26181	26139
26183	26196
26184	26215
26185	26201
26186	26182
26189	26136
26190	26084
26191	26089
26193	
26194	
26199	
26200	
26206	
26207	
26209	
26210	
26213	
26214	
26216	
26218	
26220	
26222	
26225	
26227	

26229
26230
26231
26232
26235
26239
26240
26242
26244
26245
26246

Supplementary Table 2. Primary sequencing and assembly metrics of the single molecule real-time sequencing of the eight *N. meningitidis* serogroup Y assemblies.

Sample	No. of SMRT cells	No. of reads	Coverage	Average read length (nt)
95-134	1	72 069	116	6 198
98-182	1	50 367	108	9 339
06-178	2	90 356	153	7 768
11-7	2	92 303	151	6 131
11-14	1	60 480	132	9 595
12-176	2	93 432	1 856	9 581
12-221	3	227 496	1266	18 500
12-330	2	73 979	198	6 035

Supplementary Table 3. Corrected bases (if any) in homopolymer regions using HiSeq 2000 100x2 reads mapped onto the PacBio assemblies.

Genome	Base position	Base inserted	HiSeq mapping frequency (%)	PacBio mapping frequency of the inserted base (%)	Coding region
95-134	141847	A	211/214 (99)	19/81 (23)	no
	173439	A	203/205 (99)	10/69 (14)	no
	235489	A	140/140 (100)	12/86 (14)	yes
	406026	G	190/192 (99)	13/57 (23)	no
	446097	C	169/174 (97)	6/48 (13)	yes
	447514	C	183/186 (98)	14/58 (24)	yes
	684681	T	141/147 (96)	17/76 (22)	yes
	1125476	G	181/183 (99)	14/76 (18)	yes
	1136885	G	190/192 (99)	19/78 (24)	no
	1284770	C	224/224 (100)	11/60 (18)	yes
	1775280	T	180/181 (99)	15/89 (17)	no
	1891806	A	218/228 (96)	23/81 (28)	yes
	1962865	C	216/219 (99)	13/78 (17)	yes
	2005334	G	208/213 (98)	12/61 (20)	no
98-182	689758	T	110/112 (98)	31/111 (28)	yes
11-7	1593396[a]	A	92/92 (100)	58/113 (51)	yes
	2061001	A	220/221 (100)	46/114 (40)	no
11-14	235044	T	98/98 (100)	25/178 (14)	yes
	235071	T	89/89 (100)	16/180 (9)	yes
12-330	278434	T	244/245 (100)	14/130 (11)	no
	597862	T	117/117 (100)	13/137 (9)	no
	641555	G	168/168 (100)	25/147 (17)	yes
	801279	A	146/146 (100)	31/142 (22)	yes
	1048637	C	131/134 (98)	15/119 (13)	no
	1369022	T	113/113 (100)	25/163 (15)	no
	1499760	T	221/221 (100)	24/146 (16)	yes
	1548895	A	224/225 (100)	35/152 (23)	yes
	1790958	A	224/224 (100)	43/190 (23)	yes
	1996682	T	228/230 (99)	26/131 (20)	no
	2119879	C	179/184 (97)	12/121 (10)	no

[a]Not a homopolymer region, the corrected base is in a repetitive region of a gene encoding a putative rotamase (NEIS0276).

Supplementary Table 4. Compilation of motif_summary.csv output (with QV>60) for each isolate included in the study.

**Isolate**	**Motif**	**Modification type**	**Mean modification QV**	**% Motifs detected**	**# of motifs detected**	**# of motifs in genome**	**Mean coverage**	**Partner motif**
06-178	GATC	m6A	130	96	4,104	4,256	80	GATC
12-221[a]	GATC	m6A	771	100	4,215	4,216	608	GATC
	ACACC	m6A	320	70	2,841	4,052	601	
	CGGCACGCNNNA	m4C[b]	270	20	22	109	620	
	CGNGGTAACV	unknown	253	19	17	88	608	
12-330	GATC	m6A	96	94	3,988	4,244	61	GATC
	KNNGGCTCCNNNANNNA	m4C[b]	68	75	6	8	71	
11-14	GGNNCCCGNNNKCNC	m4C[b]	88	100	6	6	78	
	GGACCCANT	m6A	91	100	5	5	76	
	GATC	m6A	113	94	3,951	4,196	70	GATC
12-176	GATC	m6A	152	96	4,043	4,204	101	GATC
	GGNNCCCNAANNCNNNNC	m4C[b]	120	90	9	10	107	
95-134	CACNNNNNTAC	m6A	93	95	429	451	63	GTANNNNNGTG
	GTANNNNNGTG	m6A	93	93	419	451	61	CACNNNNNTAC
	GATC	m6A	97	94	3,969	4,242	62	GATC
11-7	GTANNNNNGTG	m6A	121	98	439	449	79	CACNNNNNTAC
	CACNNNNNTAC	m6A	120	96	430	449	79	GTANNNNNGTG
	GATC	m6A	128	95	4,051	4,252	80	GATC
98-182	GGNNCCCGNNNGCNC	m4C[b]	74	100	5	5	57	
	CACNNNNNTAC	m6A	89	96	438	454	55	GTANNNNNGTG
	GTANNNNNGTG	m6A	89	93	423	454	56	CACNNNNNTAC
	GATC	m6A	93	94	3,960	4,202	56	GATC

[a]Isolate 12-221 was sequenced with higher coverage and QV was set to minimum 200

[b] Called as m4C but shown to be m5C by subsequent manual analysis of the data

Supplementary Table 5. Allelic differences (n=97) and their corresponding amino acid impact distinguishing the two sublineages. Putative horizontal gene transfer is marked in grey.

**Locus**	**Gene product**	**Allele no. sublineage 1**	**Product length**	**Allele no. sublineage 2**	**Product length**	**AA[a] difference**
NEIS0044	putative inner membrane transport protein	104	673	45	673	1
NEIS0047 (*rfbB*)		270	347	289	347	2
NEIS0048 (*galE*)	UDP-glucose epimerase	38	339	169	339	0
NEIS0062 (*galE2*)	truncated galE	6	233	29	233	9
NEIS0108	SMF-family protein	105	396	101	396	0
NEIS0110	DNA topoisomerase I	108	769	127	769	0
NEIS0124	DNA-directed RNA polymerase subunit beta'	151	1392	54	1392	0
NEIS0129 (*rpsJ*)	30S ribosomal protein S10	32	104	23	104	1
NEIS0137 (*rplV*)	50S ribosomal protein L22	12	110	1	110	0
NEIS0251	NADH dehydrogenase subunit L	183	675	51	675	0
NEIS0275	putative outer membrane solvent tolerance protein	108	803	112	803	0
NEIS0291 (*lot*)	LOS O-acetyltransferase	N/A[b]	599	46	623	23
NEIS0337	hypothetical protein	50	108	32	108	1
NEIS0358	acetyl-CoA carboxylase biotin carboxyl carrier protein subunit	61	159	26	151	9
NEIS0360	S-adenosylmethionine:tRNA ribosyltransferase-isomerase	63	347	45	347	5
NEIS0408 (*pilQ*)	type IV secretin protein	115	762	109	762	1
NEIS0421 (*lpxL2*)	lipid A biosynthesis lauroyl transferase	70	284	11	284	1
NEIS0425	putative peptidase	12	470	74	470	1
NEIS0524	putative peptidase	123/48	181	120/28	181	0
NEIS0550	preprotein translocase subunit SecD	116	619	55	619	0
NEIS0625	hypothetical protein	156	172	53[b]	134	65
NEIS0626	hypothetical protein	11	66	6	66	2
NEIS0628	tryptophan synthase subunit alpha	68	262	31	262	22
NEIS0647	putative amino acid permease ATP-binding protein	3	250	130	250	11
NEIS0667	hypothetical protein	1	97	124	97	7
NEIS0668	cytochrome C	1	134	125	156	8
NEIS0669 (*hemH*)	ferrochelatase	45	337	9	337	13
NEIS0671	queuine tRNA-ribosyltransferase	56	372	12	372	11

NEIS0672	threonyl-tRNA synthetase	106	644	556	638	7
NEIS0742	putative amino acid permease ATP-binding protein	129	252	62	252	0
NEIS0795	putative periplasmic protein	35	219	117	219	1
NEIS0821	putative sulphate permease inner membrane protein	152	287	20	287	1
NEIS0828 (*pilI*)	type IV biogenesis protein	16	207	198	207	1
NEIS0831 (*pilX*)	minor pilin	174	163	60	163	1
NEIS0928 (*sdhB*)	succinate dehydrogenase iron-sulfur protein (EC 1.3.99.1)	122	236	14	236	1
NEIS0948	anthranilate phosphoribosyltransferase	53	353	40	353	1
NEIS0962	putative integral membrane protein	63[c]	N/A	68[c]	N/A	0
NEIS1013	anthranilate synthase component I	104	492	105	492	1
NEIS1015 (*abcZ*)	ABC transporter ATP-binding protein	171	637	43	637	0
NEIS1059	hypothetical protein	146[c]	N/A	7	202	1
NEIS1126	ABC transporter ATP-binding protein	33	641	N/A[c]	224	N/A
NEIS1147	hypothetical protein	92	288	100	288	0
NEIS1157	bifunctional 3,4-dihydroxy-2-butanone 4-phosphate synthase/GTP cyclohydrolase II-like protein	126	364	59	364	1
NEIS1172	putative periplasmic protein	66	427	4	427	1
NEIS1174	DNA repair protein RadC	195[c]	N/A	5	226	3
NEIS1199	hypothetical protein	31	372	24	372	0
NEIS1208	putative transmembrane transport protein	91	676	30	676	1
NEIS1214	transcription-repair coupling factor	184	1371	432	1306	105
NEIS1216 (*panD*)	aspartate alpha-decarboxylase	6	128	3	128	1
NEIS1218 (*kdsA*)	2-dehydro-3-deoxyphosphooctonate aldolase (EC 2.5.1.55)	3	281	11	281	8
NEIS1219	hypothetical protein	25	147	15	147	12
NEIS1306	bifunctional N-succinyldiaminopimelate-aminotransferase/acetylornithine transaminase protein	22	398	115	398	1
NEIS1312	L-lactate dehydrogenase	8	391	107	391	0
NEIS1319 (*hscB*)	chaperone protein	15	167	1	167	1
NEIS1320	DNA gyrase subunit A	107	917	117	917	1
NEIS1370	putative integral membrane protein	44	503	99	503	1
NEIS1389	putative ferredoxin NADP+ reductase	31	260	4	260	4
NEIS1391	DNA polymerase III subunit	57	471	36	471	11

NEIS1392	hypothetical protein	86	392	7	392	1
NEIS1394	putative ferredoxin	106	499	1	499	1
NEIS1395	transketolase	148	660	63	660	2
NEIS1437	hypothetical protein	62	486	20	486	0
NEIS1475	diacylglycerol kinase	66	128	1	128	0
NEIS1513	AraC family transcription regulator	43	302	6	302	0
NEIS1546	hypothetical protein	80	395	96	395	1
NEIS1549 (*aniA*)	nitrite reductase, major outer membrane copper-containing protein	14[c]	N/A	307[c]	N/A	N/A
NEIS1634	membrane fusion protein	88	413	41	413	1
NEIS1637	hypothetical protein	69	523	79	523	1
NEIS1652	hypothetical protein	2	267	3	267	1
NEIS1705	chorismate mutase	25	377	116	377	14
NEIS1706	integral membrane efflux protein	56	460	55	460	5
NEIS1729 (*acnA*)	aconitate hydratase 1 (EC 4.2.1.3)	40	869	175	869	1
NEIS1735	putative permease	7	437	22	437	0
NEIS1780	putative ABC transporter ATP-binding protein	128	559	52	559	1
NEIS1812 (*yhbG*)	lipopolysaccharide ABC transporter	107	245	3	245	1
NEIS1886	hypothetical protein	63	181	62	181	0
NEIS1901 (*lgtB*)	lacto-N-neotetraose biosynthesis glycosyl transferase	81	276	24	276	13
NEIS1902 (*lgtA*)	lacto-N-neotetraose biosynthesis glycosyl transferase	79	349	22	349	2
NEIS1903	glycyl-tRNA synthetase subunit beta	39	688	20	688	13
NEIS1904	glycyl-tRNA synthetase subunit alpha	21	302	18	302	4
NEIS1965	putative inner membrane transport protein	279[c]	N/A	61	278	N/A
NEIS1972	phosphoribosylformylglycinamidine synthase	146	1321	162	1321	1
NEIS1983	hypothetical protein	261[c]	N/A	57[c]	N/A	0
NEIS1994	hypothetical protein	9	90	3	90	1
NEIS2080 (*rpsB*)	30S ribosomal protein S2	49	243	96	243	0
NEIS2149	UDP-N-acetylglucosamine 1-carboxyvinyltransferase	128	418	7	418	1
NEIS2155 (*lgtD*)	putative pseudogene	89	333	30	333	2
NEIS2163 (*csy*)	csy/D-glucose and sialic acid	37	1037	1	1038	1
NEIS2198 (*opcA*)	Outer membrane adhesin	18	N/A	86[c]	N/A	N/A
NEIS2415	hypothetical protein; putative phage related	96	442	95	442	0

NEIS2478	hypothetical protein	6	137	8	137	10
NEIS2479	Putative membrane protein	161[d]	302	31	339	37
NEIS2488 (*ycfA*)	conserved hypothetical integral membrane protein	8	263	33	263	0
NEIS2535	Type 1 restriction modification DNA specificity domain protein	N/A[d]	320	25	398	N/A
NEIS2567	CRISPR-associated endonuclease Cas1	1	305	3	915	1
NEIS3173 (*rte*)	hemagglutinin/hemolysin-like protein	1	775	66	643	5

[a] AA=amino acid

[b] Shorter version

[c] Premature stop codon/frameshift

[d] Insertion transposase

Supplementary Table 6. Clonal complex 23 accessory loci (n=24) and their corresponding protein functions.

**Locus**	**Gene product**
NEIS0586	MafB toxin
NEIS0599	alternative toxic C-terminal extremity
NEIS0601	putative mafS2 cassette
NEIS1344	RTX iron-regulated frpc protein outer membrane
NEIS1800	alternative toxic C-terminal extremity
NEIS1805	hypothetical protein
NEIS1979	hypothetical protein
NEIS2083	MafA3 lipoprotein
NEIS2084	MafB3 toxin protein
NEIS2514	hypothetical protein
NEIS2517	putative permease
NEIS2518	putative HKD family nuclease
NEIS2528	ferric alcalign siderophore receptor
NEIS2529	putative tonB dependent receptor
NEIS2532	putative protein containing a region found in relA/spoT proteins
NEIS2533	hypothetical protein
NEIS2534	Type I restriction-modification system R protein
NEIS2535	Type I restriction-modification system S protein
NEIS2562	cation transporter
NEIS2563	hypothetical protein
NEIS2569	hypothetical protein
NEIS2570	ATPase containing AAA domain (ATPases Associated with diverse cellular Activities)
NEIS2571	VWA domain protein interacting with AAA ATPase

NEIS2572	hypothetical protein

Supplementary Table 7. Genes putatively associated with pathogenic interactions absent in all sublineage 1 and 2 genomes.

**Gene**	**Product/function**	**Size (nt[a])**
hmbR	Haemoglobin binding protein	2361-2477
NEIS0444 *(tpsA)*	Hemagglutinin	6081
NEIS1194 (*modB*)	Type III restriction modification system methylase	1909-2114
NEIS1454	Virulence associated protein	N/A
NEIS1866 (*tspB*)	TspB protein	1482-1650
NEIS1969 (*nadA*)	Adhesion/invasion (NMB1994)	750-2064
NEIS2012 (*lpt6*)	O-6 PEA LOS[b] transferase	1553-1664
NEIS2362	NgoAV Type I restriction modification system, specificity subunit S	600-1260
NEIS2364 (*modD*)	Type III restriction modification system methylase	1903-2050
NEIS2538	Uncharacterized homolog of phage Mu protein	N/A
NEIS2849	Bacteriophage Mu Gp45 protein	630
NEIS2915	Phage Mu protein F like protein	1347

[a] nt=nucleotide

[b] LOS=lipooligosaccharide

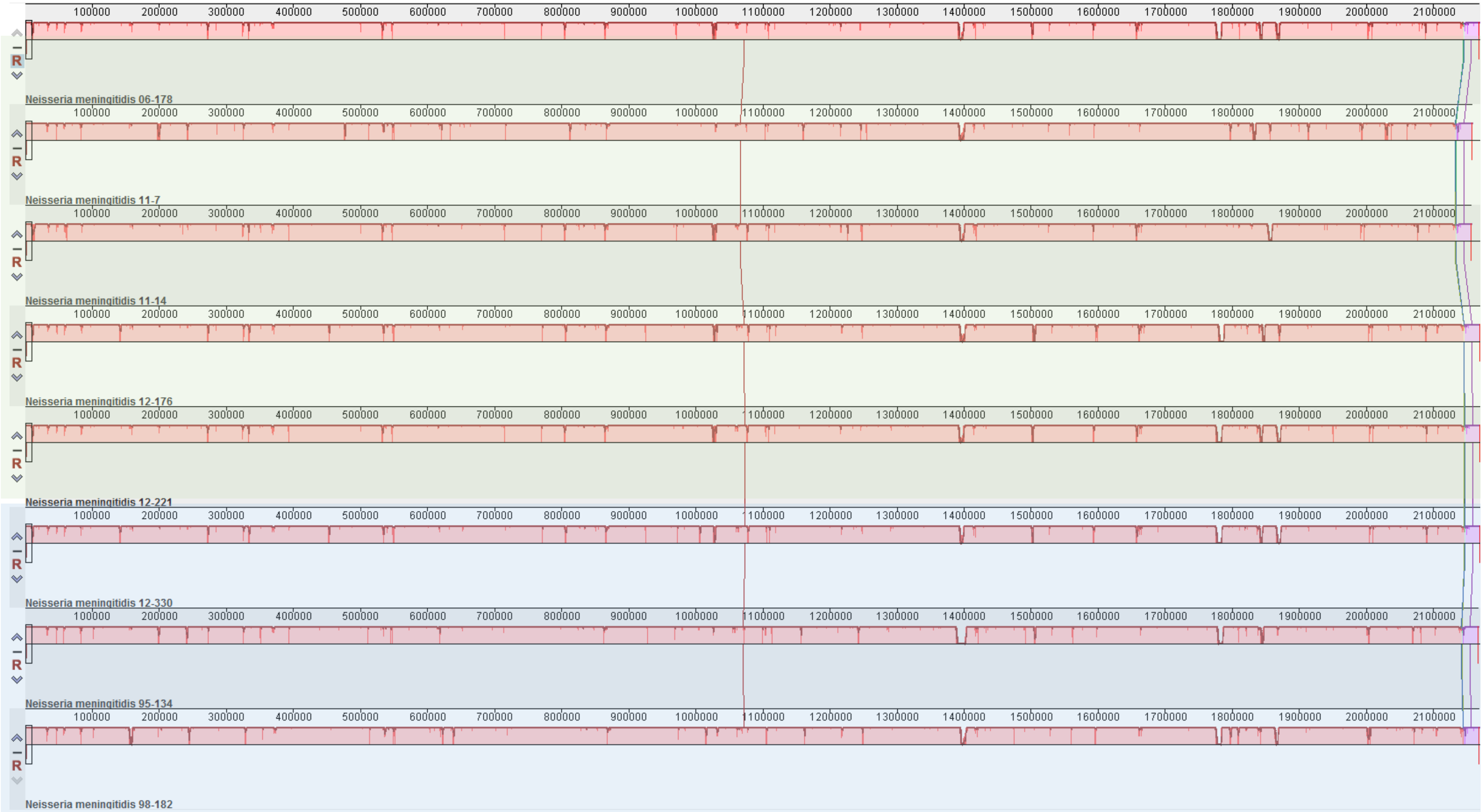

Supplementary Figure 1. Multiple genome alignment of *N. meningitidis* serogroup Y genomes grouped by sublineage: sublineage 1 (n=5) in green box and sublineage 2 in blue box (n=3).
